# Neurofilament heavy polypeptide protects against reduction in synaptopodin expression and prevents podocyte detachment

**DOI:** 10.1038/s41598-018-35465-6

**Published:** 2018-11-21

**Authors:** Juan Wang, Teruo Hidaka, Yu Sasaki, Eriko Tanaka, Miyuki Takagi, Terumi Shibata, Ayano Kubo, Juan Alejandro Oliva Trejo, Lining Wang, Katsuhiko Asanuma, Yasuhiko Tomino

**Affiliations:** 1 Division of Nephrology, Department of Internal Medicine, Juntendo University Faculty of Medicine, Hongo 2-1-1, Bunkyo-ku, Tokyo, 113-8421 Japan; 2 Department of Nephrology, Chiba University Graduate School of Medicine, 1-8-1 Inohana, Chuo-ku, Chiba, 260-8670 Japan; 3 Department of Nephrology, the First Hospital of China Medical University, No.155 NanjingBei Street, Shenyang, Liaoning 110001 China; 4 Department of Pediatrics and Developmental Biology, Graduate School of Medicine, Tokyo Medical and Dental University, Yushima 1-5-45, Bunkyo-ku, Tokyo, 113-8510 Japan; 5 Department of Cellular and Molecular Neuropathology, Juntendo University Graduate School of Medicine, Hongo 2-1-1, Bunkyo-Ku, Tokyo, 113-8421 Japan; 6 Asian Pacific Renal Research Promotion Office, Medical Cooporation SHOWAKAI, Tokyo, Japan

## Abstract

Podocytes are highly specialized cells that line the glomerulus of the kidney and play a role in filtration. Podocyte injury plays a critical role in the development of many kidney diseases, but the underlying mechanisms remain unclear. In this study, we identified that neurofilament heavy polypeptide (NEFH), an intermediate filament component, protects podocyte from injury. We observed that NEFH was upregulated after ADRIAMYCIN(ADR)-induced podocyte injury in both mice and cultured murine podocytes. Immunofluorescence and co-immunoprecipitation analyses revealed that NEFH was colocalized with synaptopodin, a podocyte-specific marker. High NEFH expression in podocytes prevented the Adriamycin-induced reduction in synaptopodin expression. The siRNA-mediated knockdown of NEFH in podocytes reduced the number of vinculin-containing focal contacts, thereby reducing adhesion to the extracellular matrix and increasing podocyte detachment. In addition, NEFH expression was significantly increased in renal biopsy specimens from patients with focal segmental glomerulosclerosis and membranous nephropathy, but in those with minimal change disease. These findings indicate that NEFH is expressed in podocytes during the disease course and that it prevents the reduction in synaptopodin expression and detachment of podocytes.

## Introduction

There is abundant evidence, both from animal experimental models and human studies, that podocyte injury is involved in many kidney diseases. Disruption of podocyte structure or function leads to proteinuria and exacerbation of renal function, characterized histologically by foot process (FP) effacement, podocyte loss, and glomerulosclerosis. The mechanisms of podocyte injury have been widely studied using ADRIAMYCIN(ADR)-induced renal damage models. In a mouse ADR-induced nephropathy model that mimics the nephropathy seen in human focal segmental glomerulosclerosis (FSGS)^1^, FP effacement and detachment from the glomerular basement membrane (GBM) cause proteinuria and the attenuation of glomerular filtration function^2,3^ We have previously suggested that ADR treatment leads to actin microfilament rearrangement and podocyte apoptosis *in vitro*^3^. However, the downstream signaling process in ADR-injured podocytes remains unclear.

In this study, we used the ADR-induced podocyte injury model to elucidate the underlying mechanisms of podocyte injury and to explore novel candidate genes related to this process. We performed a microarray assay on ADR-treated podocytes and surprisingly found that the neurofilament heavy polypeptide (*NEFH*) gene, which encodes NEFH—a type IV intermediate filament (IF), was significantly upregulated after ADR treatment, despite its expression being confined to neurons. Therefore, we investigated the involvement of NEFH in podocyte injury, including its function in severe podocyte injury.

## Results

### ADR increased NEFH expression in cultured podocytes

**Figure 1:**
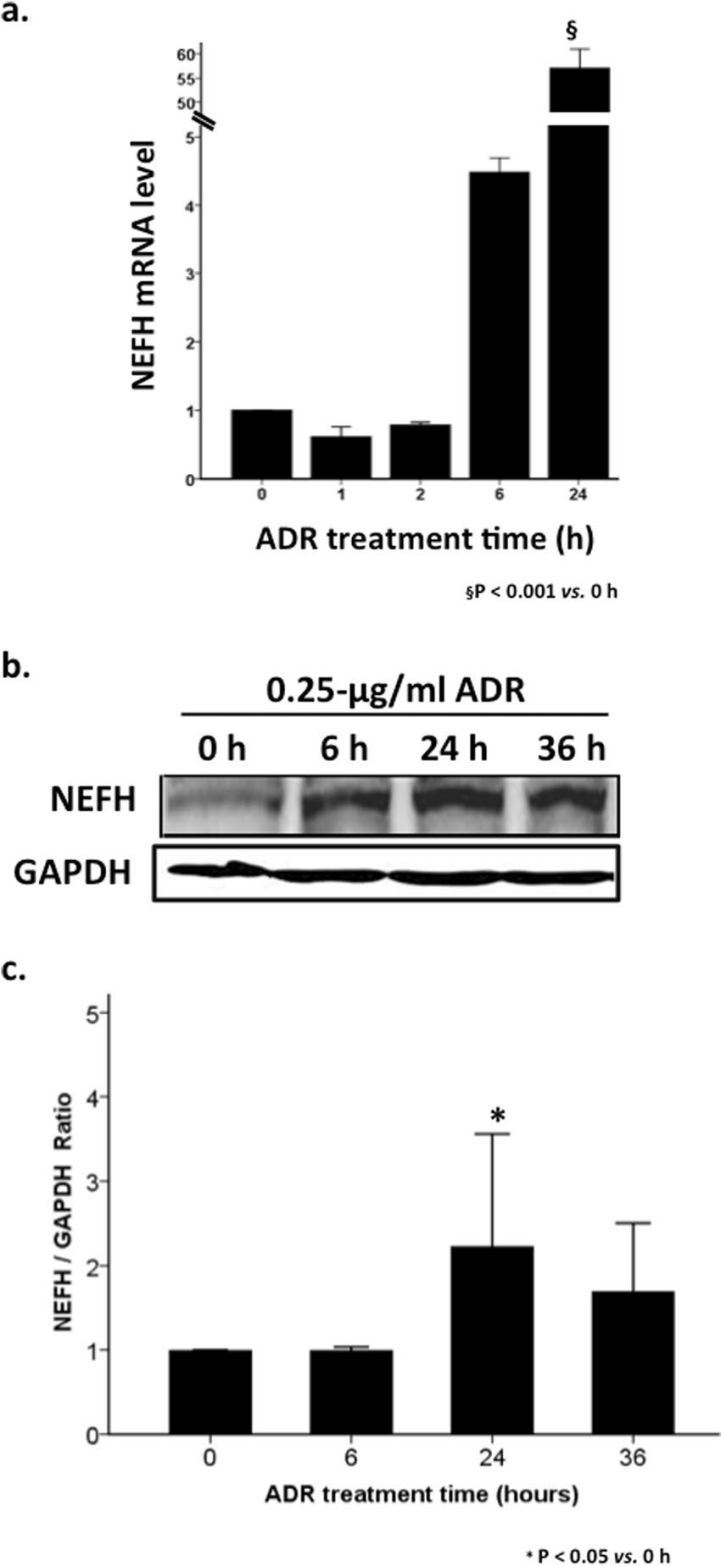
Adriamycin^®^ (ADR) treatment induced neurofilament heavy polypeptide (NEFH) upregulation in cultured podocytes. Upregulation of NEFH expression in mouse podocytes induced by 0.25 μg/ml ADR was confirmed at both mRNA (**a**) and protein levels (**b,c**). (**a**) ^§^P < 0.001, 24 h vs. 0 h; n = 3. Data are presented as mean ± SD (**c**) Quantification of NEFH expression. *P < 0.05, 24 h vs. 0 h; n = 4. Data are presented as mean ± SE. GAPDH, glyceraldehyde-3-phosphate dehydrogenase.

To investigate whether novel molecules were involved in ADR-induced podocyte injury, a microarray assay was performed on cultured podocytes with or without treatment with 0.25 μg/ml of ADR. Among the genes that were significantly expressed in the ADR-treated podocytes, *NEFH* was extremely upregulated, especially at 24 h after ADR treatment, with a 35.82-fold increase compared to the level in untreated podocytes (Table 1). Its expression was confined to neurons. To confirm this significant finding, NEFH mRNA level and protein expression were measured in differentiated podocytes treated with 0.25 μg/ml of ADR and in untreated podocytes. ADR treatment resulted in a significant increase (55.98-fold) in the NEFH mRNA level (Fig. 1a) and a 2.4-fold increase in protein expression (Fig. 1b,c) at 24 h compared to the values in untreated podocytes.

**Table 1:** Neurofilament heavy polypeptide (NEFH) expression in the microarray assay.

Gene symbol	Entrez Gene	Fold change (hours after treatment with 0.25-μg/ml ADR)				
		0 h	1 h	2 h	6 h	24 h
*NEFH*	380684	1.00	1.19	1.71	7.58	35.8

A microarray assay was performed on podocytes with 0.25-μg/ml ADRIAMYCIN (ADR) treatment for 1, 2, 6, and 24 h and on untreated podocytes (0 h). Gene expression is represented as a fold-change normalized according to the level in untreated podocytes. *NEFH* (Entrez Gene for mice, 380684), which encodes neurofilament heavy polypeptide, showed significantly increased expression after ADR treatment, especially at 24 h, when there was a 35.8-fold increase compared with the expression level in untreated podocytes.

The original data will be stored in the National Center for Biotechnology Information’s Gene Expression Omnibus at https://www.ncbi.nlm.nih.gov/geo/.

### NEFH upregulation in ADR-induced nephrosis mice

**Figure 2:**
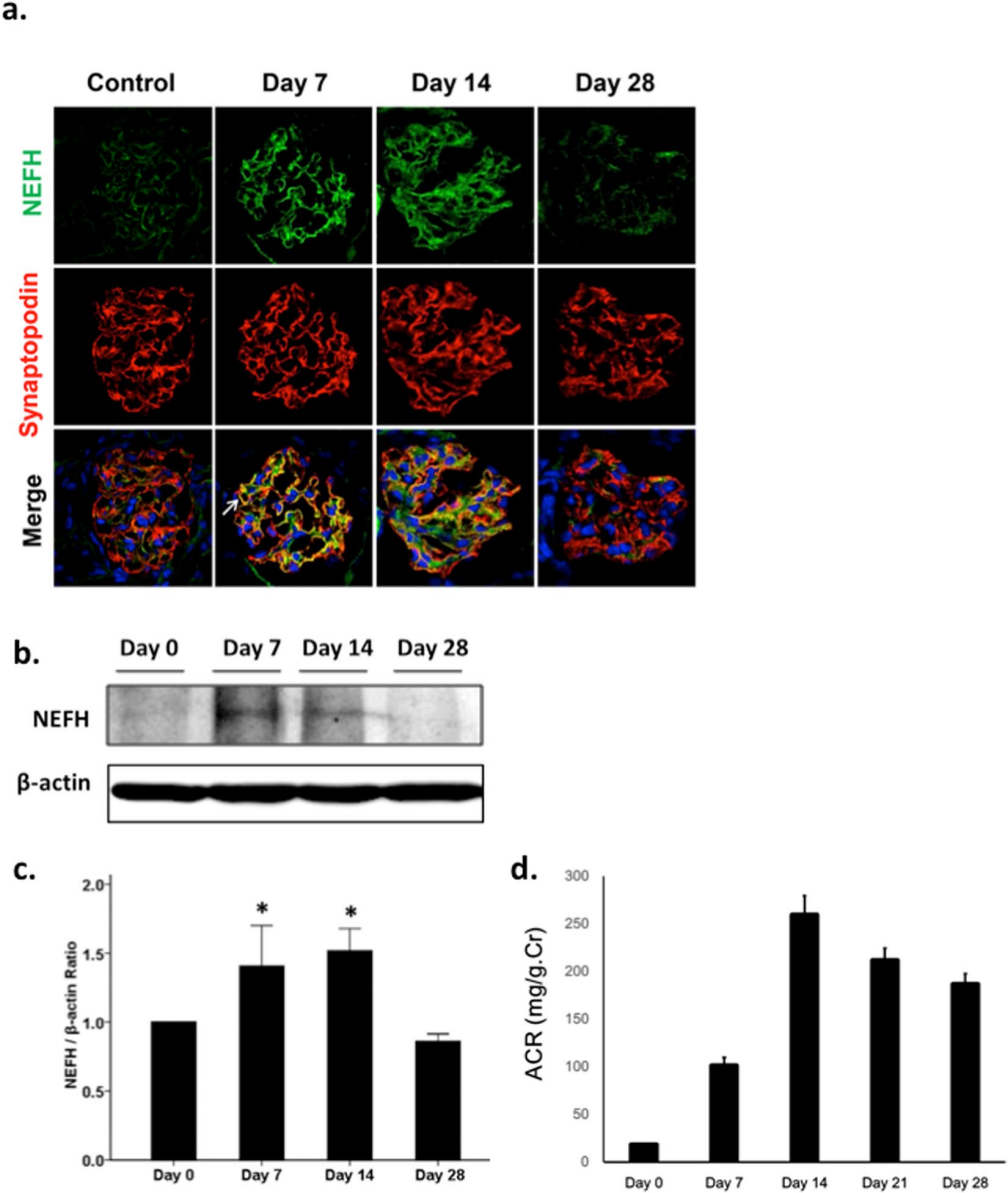
The podocytes of ADR-induced nephrosis mice showed increased NEFH expression. The NEFH expression in podocytes was examined in the kidneys of ADR-induced nephrosis mice. (**a**) Triple staining of NEFH (green), synaptopodin (red) for podocytes, and DAPI (blue) for nuclei was performed on ADR-induced nephrosis mouse kidney specimens. In normal mouse kidneys (injected with saline only, on day 0), NEFH was observed as weak linear reactivity along the glomerular capillary loops. After the ADR injection, staining showed marked increases in NEFH expression on days 7 and 14, with close overlap of the distributions of NEFH and synaptopodin (arrow in day 7). Scale bar: 20 μm, n = 4–5. (**b**) Western blot analysis of isolated glomeruli from control (day 0) and ADR-injected mice. NEFH showed significantly increased reactivity on days 7 and 14. β-actin was used as an internal control. (**c**) Representative quantification of NEFH. Data are presented as mean ± SE. *P < 0.05, day 7 vs. day 0 and day 14 vs. day 0; n = 6. (**d**) Proteinuria was shown from day 3 and it elevated peak at day 14, and it was decreased day by day until day 28. n = 6).

To detect NEFH expression *in vivo*, immunofluorescence and western blot analyses were performed on ADR-induced nephrosis kidney specimens at various time points (Fig. 2). In the normal mouse kidney, NEFH showed weak linear reactivity along the glomerular capillary loops. In the mice with ADR-induced nephrosis, proteinuria was observed from day 3 after ADR injection, with the proteinuria increasing from day 7 and reaching a peak at day 14 (Fig. 2d). Staining showed markedly increased NEFH expression on days 7 and 14 after ADR injection. The distribution of NEFH closely overlapped that of synaptopodin, a podocyte-specific marker, indicating that NEFH was localized in the podocyte FPs (Fig. 2a).

To evaluate the ADR- induced NEFH expression in mice, glomeruli were isolated from control and ADR-injected mice. Western blot analyses of glomerular lysates (Fig. 2b,c) showed significantly increased NEFH expression on days 7 and 14 after ADR injection, which was consistent with the results of the immunofluorescence analyses (Fig. 2a). These findings suggested that NEFH upregulation in podocytes was accompanied by the initial appearance of proteinuria in ADR-induced renal injury^3,4^.

### Specimens from patients with FSGS or membranous nephropathy (MN), but not minimal change disease (MCD), showed elevated NEFH

**Figure 3:**
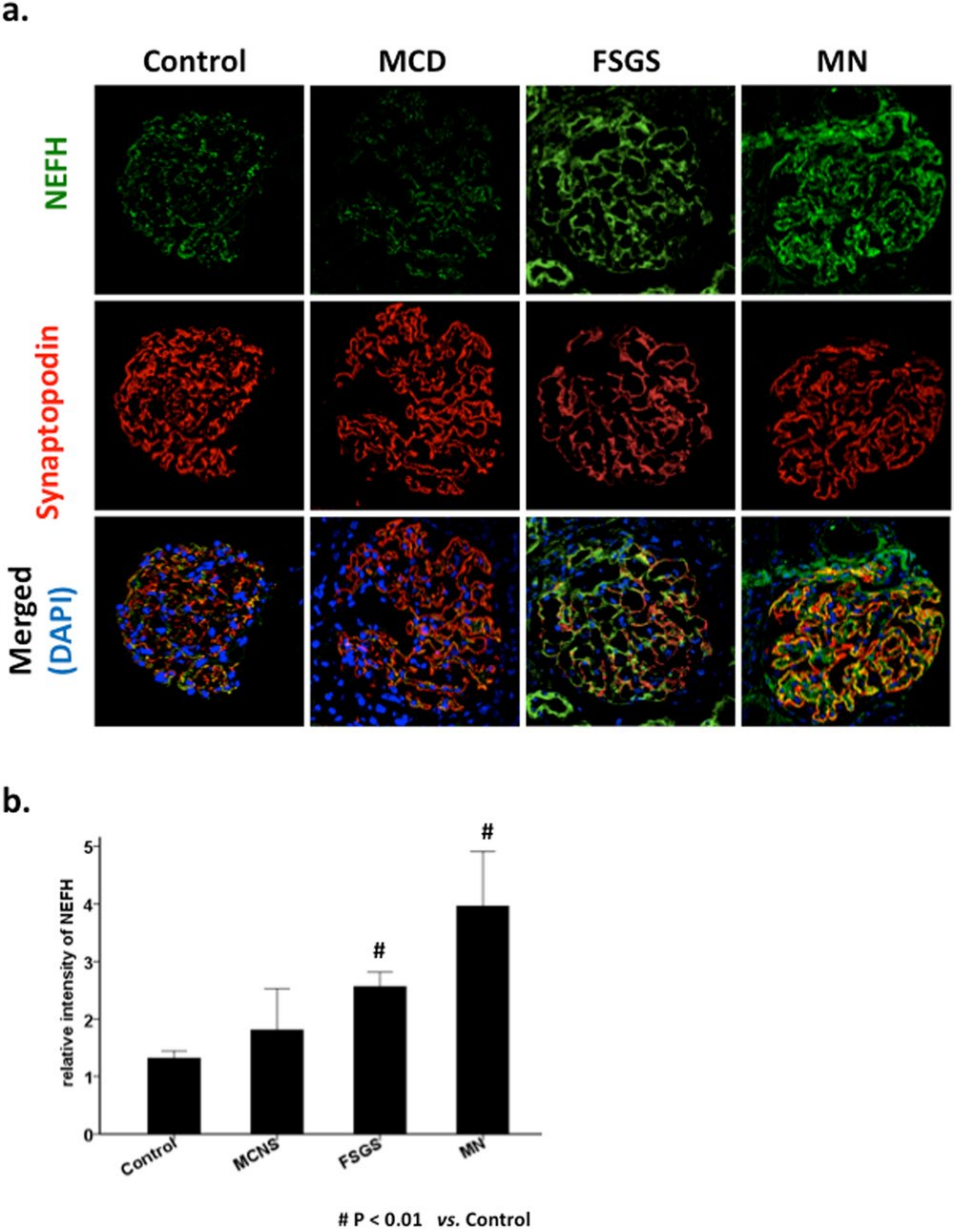
NEFH expression was significantly increased in human focal segmental glomerulosclerosis (FSGS) and membranous nephropathy (MN), but not in minimal change disease (MCD). Renal biopsy sections from several human nephropathies were stained for NEFH (green), synaptopodin (red), and DAPI (blue). Samples from patients with minor glomerular abnormalities were used as controls (n = 7). (**a**) Low NEFH expression was observed in the MCD samples (n = 4), similar to that observed in the controls; much higher expression was observed in the MN (n = 4) and FSGS (n = 11) samples, showing linear overlapping with the synaptopodin signal. Scale bar: 20 μm. (**b**) Representative fluorescence intensity of NEFH. Data are presented as mean ± SE. ^#^P < 0.01, FSGS vs. control and MN vs. control; n = 4–11.

To examine NEFH expression in human kidney diseases, human kidney biopsy specimens were stained with anti-NEFH antibodies (Fig. 3a). All cases were clinically diagnosed as nephrotic syndrome or glomerulonephritis (Supplementary Table 1), with MCD, FSGS, or MN, according to the biopsy findings. Samples from patients with minor glomerular abnormalities were used as controls. Staining showed that NEFH expression was weak in MCD, similar to that observed in the control, but was strong in FSGS and MN, showing a linear overlapping signal with synaptopodin expression. The fluorescence intensity of NEFH was analyzed, revealing a significant increase in NEFH expression in the samples from patients with FSGS and MN, but not in those from patients with MCD (Fig. 3b).

### Gene interference of NEFH disrupted podocyte morphology

**Figure 4:**
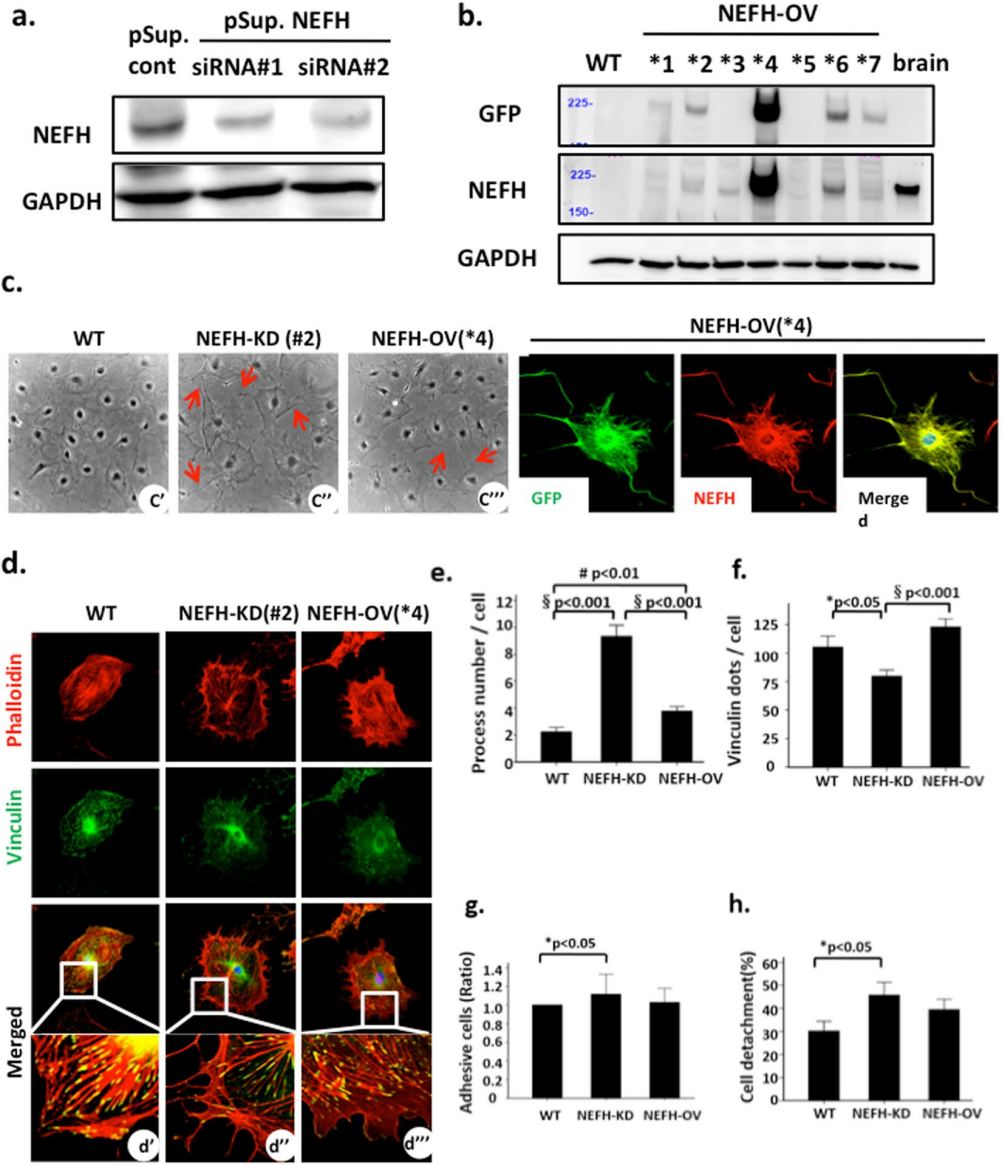
Morphologic changes to podocytes with NEFH knockdown (NEFH-KD) and NEFH overexpression (NEFH-OV). (**a**) Western blot analysis showed that NEFH was suppressed in podocytes using two different NEFH siRNAs (pSup.NEFH siRNA#1 and #2), but not with control siRNA with a scrambled sequence (pSup.cont). (**b**) Seven cloned NEFH-OV podocyte cell lines (NEFH-knock-in) were verified with separate western blotting for NEFH and green fluorescent protein (GFP), with a mouse brain sample used as a positive control (upper panel). The bottom panel shows merged imaging for the NEFH and GFP in NEFH-OV podocytes, demonstrating their closely overlapping distributions. Original magnification ×400. (**c**) Representative images of podocyte morphology under phase contrast microscopy (c’,c”,c”’). Gene silencing of NEFH [NEFH-KD (#2), c”] and exogenous NEFH [NEFH-OV (*4), c”’] in podocytes resulted in the formation of longer processes (arrows) than those in the wild-type (WT) podocytes (c’). Original magnification ×100. (**d**) WT, NEFH-KD, and NEFH-OV podocytes were triple stained for phalloidin (red) for F-actin, vinculin (green) for focal contacts, and DAPI (blue). Vinculin dots show positive staining at the edge of the actin filaments (yellow; the lowest two lanes, with magnified images). (**e**) The process number per cell significantly increased in the NEFH-KD and NEFH-OV podocytes. ^§^P < 0.001, ^#^P < 0.01; n > 30 cells from nine separate culture wells in each group. (**f**) The number of vinculin dots per cell was significantly lower in the NEFH-KD podocytes than in the WT or NEFH-OV podocytes. *P < 0.05, ^§^P < 0.001; n > 30 cells from nine separate culture wells were counted in each group. (**g**) Adhesion study. NEFH-KD reduced the ability of podocytes to adhere to the substrate. *P < 0.05; n = 9. (**h**) Detachment study. NEFH-KD increased podocyte detachment induced by ADR treatment. *P < 0.05; n = 9.

To elucidate the function of NEFH in podocytes, we established stable NEFH-knockdown (NEFH-KD) podocytes using the pSuperRNAi system. Using western blotting, we confirmed that two different NEFH siRNAs (pSup.NEFH siRNA#1 and #2), but not control siRNA (pSup.cont) with a scrambled sequence, suppressed NEFH expression in cultured podocytes (Fig. 4a). Next, we developed stable NEFH-overexpressed (NEFH-OV) podocytes through transfection of the green fluorescent protein (GFP)-NEFH construct into cultured podocytes. The lysates of NEFH-OV podocytes were checked using western blotting for GFP and NEFH reactivity separately (Fig. 4b, upper panel). The cloned cells positive for both GFP and NEFH indicated successful generation of NEFH-OV podocytes, and we selected clone *4, which exhibited the highest NEFH expression, for subsequent experiments. We also confirmed NEFH-OV podocytes with GFP and NEFH immunofluorescence, showing a closely colocalized image (Fig. 4b, bottom panel). Using phase contrast microscopy, we investigated changes in cellular morphology and observed the formation of many processes in both NEFH-KD and NEFH-OV podocytes (Fig. 4c). Because podocytes contain actin, especially in FPs in the glomeruli, we performed a double immunofluorescence analysis with phalloidin and vinculin, a component of focal contact that is closely associated with actin filaments (AFs). In the NEFH-KD podocytes, phalloidin showed positive staining in the process formation (Fig. 4d). In a quantitative experiment, we counted the number of actin-positive processes in NEFH-KD, NEFH-OV, and wild-type (WT) podocytes, using the WT podocytes as a control because no morphological difference was observed between WT and pSup.cont podocytes. We found significantly greater process formation in the NEFH-KD podocytes than in the WT and NEFH-OV podocytes (P < 0.001, NEFH-KD vs. WT; P < 0.001, NEFH-KD vs. NEFH-OV; Fig. 4e). Furthermore, double staining for phalloidin and vinculin demonstrated overlapping reactivity at the end of stress fibers, with much less focal contact, i.e., smaller yellow dots, in the NEFH-KD podocytes than in the WT or NEFH-OV podocytes (Fig. 4d). An immunofluorescence intensity analysis showed a significant decrease in the number of vinculin dots in the NEFH-KD podocytes (P < 0.05, NEFH-KD vs. WT; P < 0.001, NEFH-KD vs. NEFH-OV; Fig. 4f).

Because podocytes anchor to the GBM through focal contact and integrin family proteins, we investigated the alternation of adhesive and detachment potentials of podocytes induced by *NEFH* gene silencing. The knockdown of NEFH reduced the podocyte adhesive potential to the extracellular matrix under normal culture conditions (P < 0.05, compared with WT; Fig. 4g). With ADR-induced injury, the NEFH-KD podocytes showed an increased number of detached cells compared to the WT podocytes (P < 0.05; Fig. 4h). However, no significant difference was observed in adhesive ability or detachment properties between the NEFH-OV and WT podocytes. These findings suggested that the knockdown of NEFH resulted in diminished focal contact with the extracellular matrix, allowing detachment from the GBM to be easily induced. Adhesion and detachment assays comparing WT and pSup.cont podocytes showed no difference between them.

### NEFH was colocalized with vimentin and nestin in NEFH-overexpressed podocytes and was partially colocalized with them in ADR-treated kidney specimens

**Figure 5:**
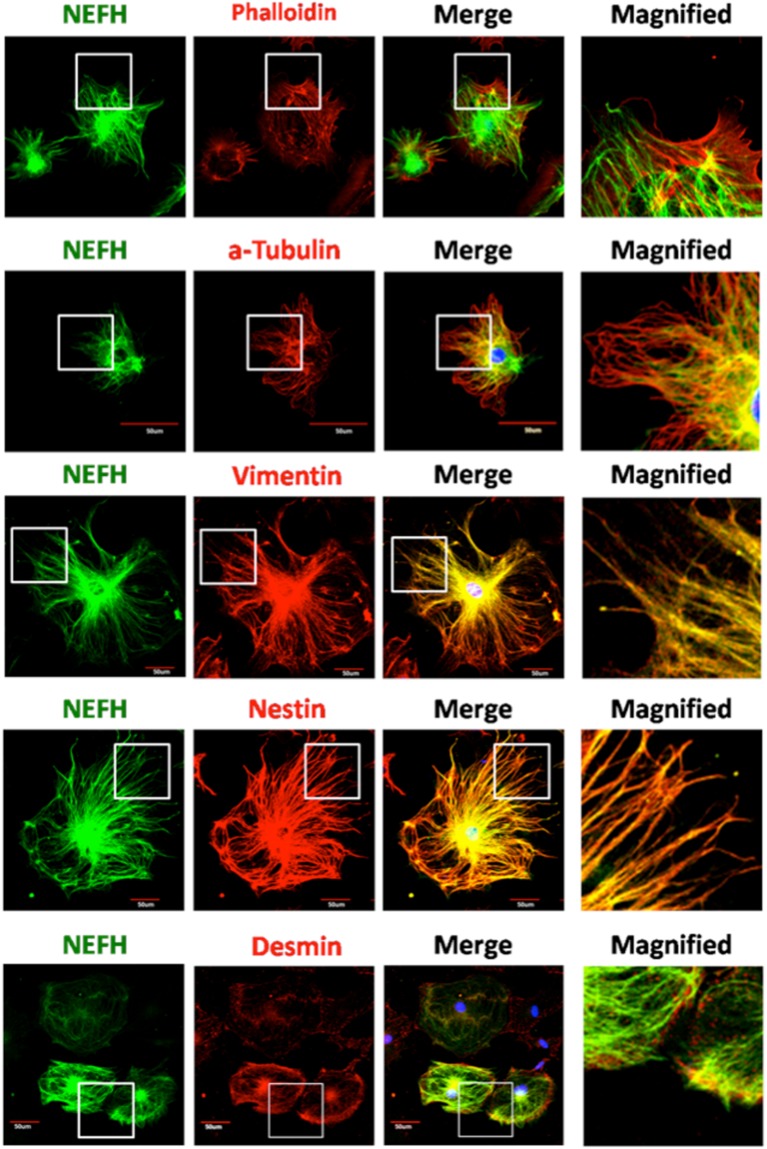
NEFH was colocalized with vimentin and nestin in NEFH overexpression (NEFH-OV) podocytes. The relationship between NEFH and other cytoskeletal proteins was confirmed with double staining of NEFH-OV podocytes. Phalloidin was stained for actin filaments, α-tubulin for microtubules, and vimentin, nestin, and desmin for intermediate filaments. The actin filaments were thicker and denser in the NEFH-OV podocytes than in the wild-type podocytes. NEFH was closely colocalized with vimentin and nestin in the podocytes, but not with α-tubulin and desmin. Original magnification ×400.

**Figure 6:**
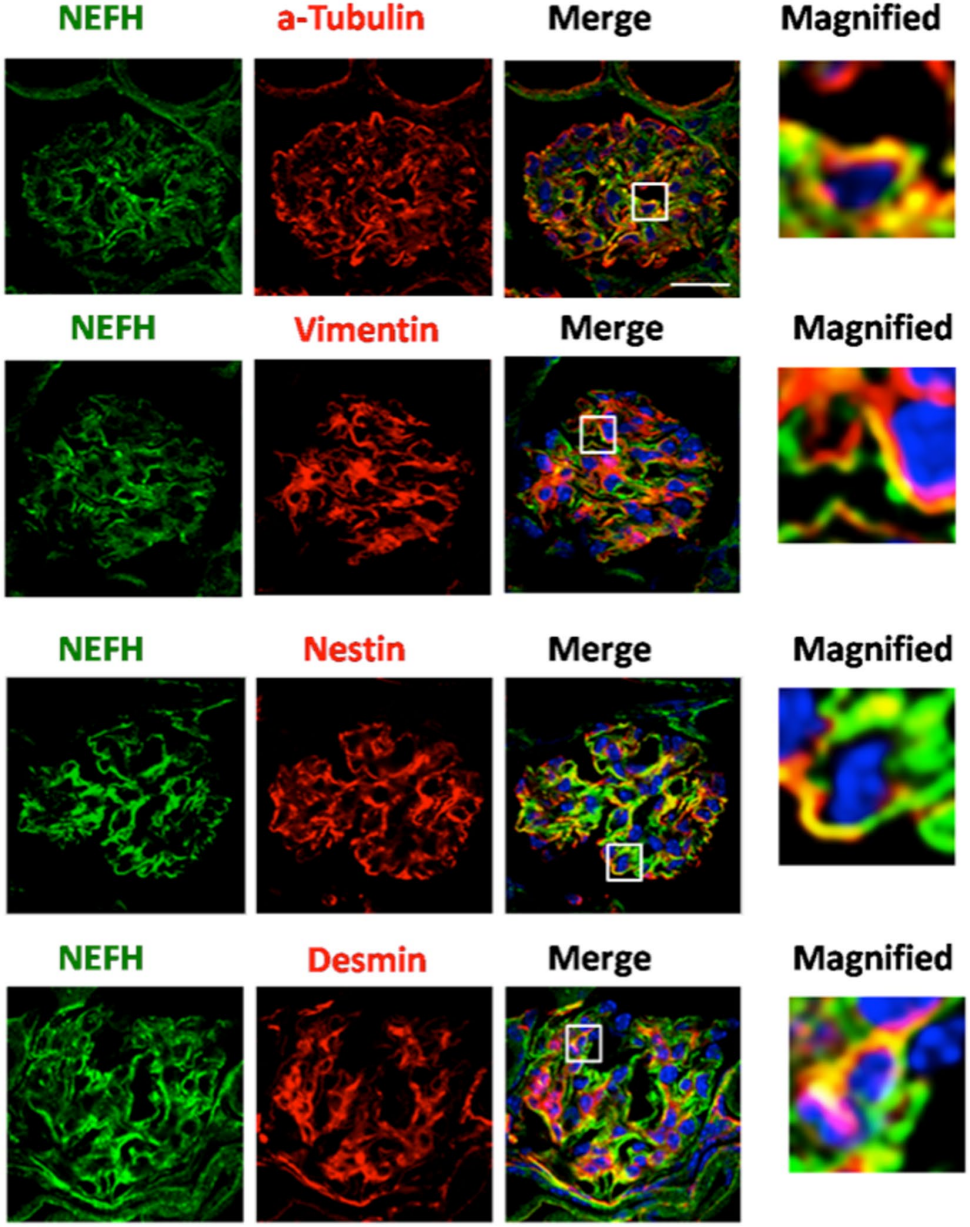
NEFH was partially colocalized with vimentin and nestin in ADR-induced nephrosis mice kidneys. The kidney specimens obtained from ADR-induced nephrosis mice were stained for NEFH (green) and α-tubulin, vimentin, nestin, or desmin (red) as well as for nuclei (blue) with DAPI. NEFH was partially colocalized with α-tubulin, vimentin, nestin, and desmin. Scale bar: 20 μm; n = 4–5.

We performed immunostaining of NEFH with α-tubulin and vimentin, nestin, or desmin in NEFH-OV podocytes (Fig. 5) and kidney specimens on day 3 after ADR-induced injury (Fig. 6) because control mice (treated only with saline) showed only little NEFH expression and we had previously reported that podocyte injury starts on day 3 with dendrin expression and apoptotic changes^3^. To compare the interaction among IFs, we performed *in vitro* experiments using NEFH-OV podocytes. Interestingly, NEFH was closely colocalized with vimentin and nestin (Fig. 5), but only partially colocalized with them in the ADR-injured kidney specimens (Fig. 6). Neither α-tubulin nor desmin were closely colocalized with NEFH, especially in cultured NEFH-OV podocytes (Fig. 5). For comparison, similar IF staining was performed in cultured WT podocytes. In these podocytes, NEFH was not colocalized with α-tubulin, vimentin, nestin, or desmin (Supplementary Figure 1). In *in vivo* assays, α-tubulin, vimentin, nestin, and desmin were only partially colocalized with NEFH (Fig. 6).

### NEFH overexpression in podocytes rescued ADR-induced reduction in synaptopodin expression

**Figure 7:**
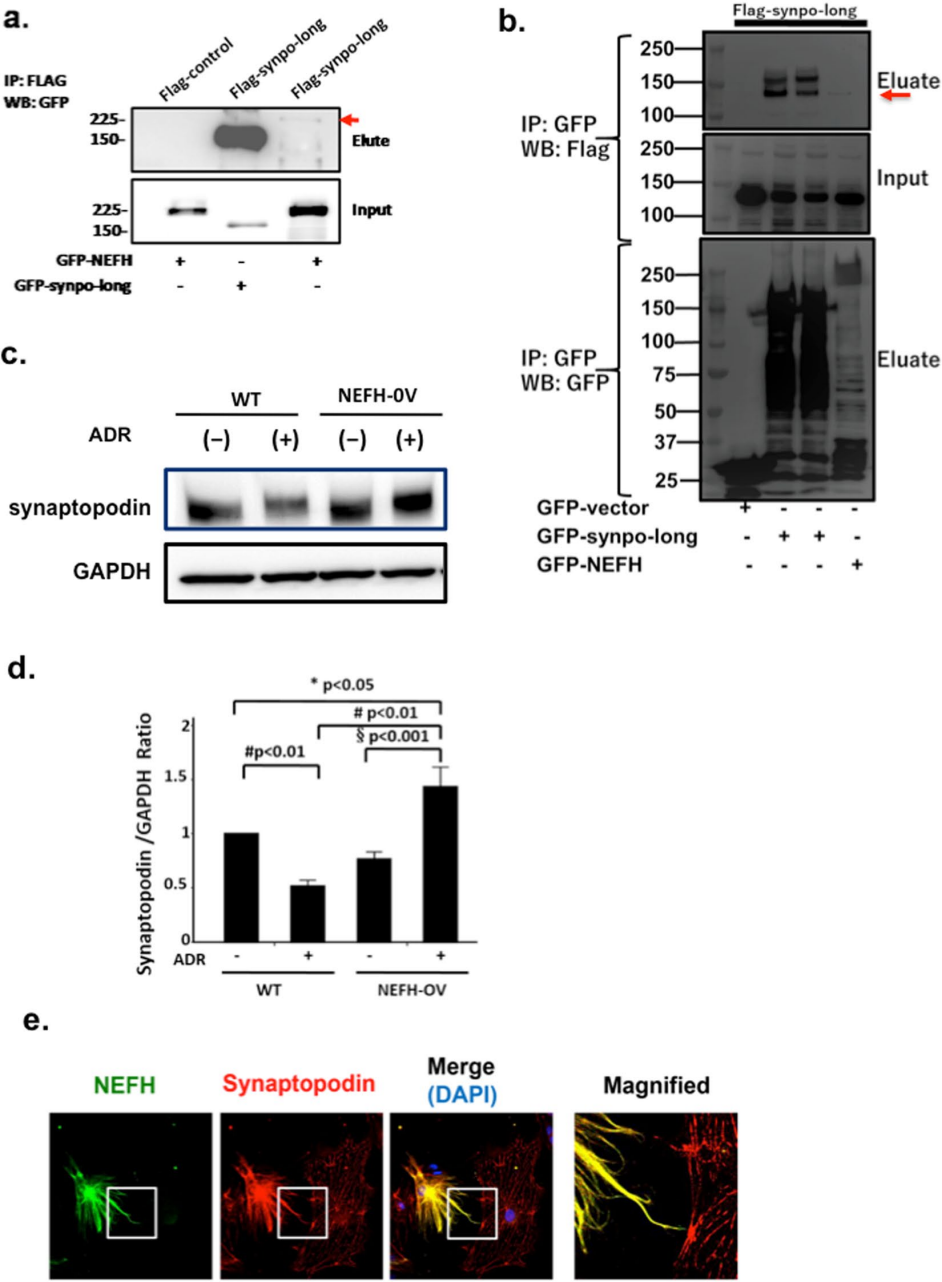
NEFH overexpression in podocytes rescued the ADR-induced induced reduction in synaptopodin expression. (**a**) Co-immunoprecipitation findings suggested that exogenous NEFH and synaptopodin were co-assembled in HEK293 cells (arrow). (**b**) Co-immunoprecipitation with GFP beads confirmed the exogenous interaction with synaptopodin and NEFH (arrow) (**c**) Western blotting showed that synaptopodin expression increased in NEFH-overexpressed (NEFH-OV) podocytes at 24 h but decreased in wild-type (WT) podocytes (upper lane). GAPDH was used as an internal control. (**d**) Quantification of the synaptopodin expression. Data are presented as mean ± SE. *P < 0.05, ^#^P < 0.01, ^§^P < 0.001; n = 6 culture dishes. (**e**) Immunofluorescence of synaptopodin in the NEFH-rescue experiment through transfection of a GFP–NEFH construct into ADR-treated podocytes. After exposure to ADR, the immunoreactivity of synaptopodin (red) was preserved in podocytes with high NEFH expression (green; right cell), which exhibited intense filamentous imaging, but not in podocytes with low NEFH expression (left cell), which showed weakly expressed dotted imaging. Original magnification ×400. NEFH, neurofilament heavy polypeptide; ADR, Adriamycin^®^; GFP, green fluorescent protein; GAPDH, glyceraldehyde-3-phosphate dehydrogenase.

In ADR-injured mouse kidneys and human kidney biopsy specimens, we observed that increased NEFH expression was associated with closely colocalized immunoreactivity with synaptopodin. Therefore, we hypothesized that there is an interaction between NEFH and synaptopodin. To test this, we performed an exogenous co-immunoprecipitation study in HEK293T cells. The results showed that NEFH co-assembled with synaptopodin (Fig. 7a). To further confirm their interaction, we performed an exogenous co-immunoprecipitation assay in the reverse direction (Fig. 7b). Both these experiments demonstrated that synaptopodin binds with NEFH. Next, we checked the endogenous synaptopodin levels in NEFH-OV podocytes. Under normal conditions, both WT and NEFH-OV podocytes showed a strong synaptopodin signal. At 24 h after ADR treatment, synaptopodin expression increased in the NEFH-OV podocytes but decreased in the WT podocytes (Fig. 7c,d). To further confirm the protective role of NEFH on synaptopodin under ADR exposure, we performed a NEFH-rescue experiment involving the transfection of an exogenous GFP–NEFH construct into ADR-treated podocytes. Consistent with our hypothesis, the immunofluorescence of synaptopodin was preserved in podocytes that highly expressed NEFH, showing intense filamentous imaging, in contrast to weakly expressed dotted imaging in podocytes with low NEFH expression (Fig. 7e).

## Discussion

In this study, we identified NEFH as a new IF that was upregulated in ADR-injured podocytes and in human glomerular diseases (FSGS and MN). NEFH protects podocytes by stabilizing synaptopodin. NEFH is a component of neuronal cytoskeleton neurofilaments (NEFs), which comprise internexin and three subunits named according to their molecule weight: NEFH, neurofilament medium polypeptide (NEFM), and neurofilament light polypeptide (NEFL)^5^. In neurons, NEFH co-assembles with NEFM and NEFL to form a heteropolymer filament and combines with other IFs, microtubules (MTs), and actin microfilaments directly or via cross-bridging proteins^5,6^. NEFs are responsible for mediating radial growth, a process that determines the axonal diameter. NEFs are phosphorylated on highly conserved lysine–serine–proline (KSP) repeats located along the C-termini of both NEFM and NEFH within the myelinated axonal regions^1,7–9^. Phosphorylation is thought to regulate NEF transport and function. KSP phosphorylation is essential for radial growth and suggests an alternative role for NEF phosphorylation in myelinated axons^10^. In the present study, NEFH was upregulated in injured podocytes, and phosphorylated NEFH (pNEFH) was also upregulated in a similar pattern (data not shown). Both NEFH and pNEFH were expressed similarly, and no difference was detected between them. The role of NEFH in podocytes appears to differ from its role in neurons because of differences in function and structure. Hence, in this study, we focused on NEFH, and but not pNEFH, in podocyte injury.

Our microarray data revealed *NEFH* expression in ADR-treated podocytes was upregulated by 35.8-fold compared with that in untreated podocytes. In contrast, the NEFH protein level was upregulated by 2.4-fold in ADR-treated podocytes compared with the level in untreated podocytes. This difference during microarray and protein expression in upregulation may be related to the balance of production and degradation system. Despite this difference, the protein level continues increasing substantially, indicating that abundant NEFH was produced and that it played a role in injured podocytes. NEFH was expressed transiently in the *in vivo* and *in vitro* assays; this transient expression may be associated with the single ADR injection, which initially caused drug toxicity and stress. A further study is warranted to analyze this discrepancy.

The three cytoskeletal subsystems, namely AFs, MTs, and IFs, can interact through nonspecific steric interactions. These three polymers have rather different structural and physical properties, enabling specific cellular functions^11^. Although the human IF family encompasses more than 65 different members, only vimentin and keratin have been extensively investigated. Murray *et al*.^12^ demonstrated that vimentin solubility is sensitive to perturbations in the actin and MT cytoskeleton. Esue *et al*.^13^ demonstrated that a direct interaction between actin and vimentin filaments is mediated by the tail domain of vimentin. Helfand *et al*.^14^ showed that vimentin IF at the periphery of fibroblasts inhibits the formation of lamellipodia, whereas the disassembly and withdrawal of vimentin IF from the cell periphery facilitate actin-based protrusion of the lamellipodia. Various cross-linking protein complexes, including both active components (such as actin fiber-based and MT-based motor proteins) and passive components (such as plectins and members of the plakin family) have also been extensively investigated^15^. In particular, plectin has been shown to be distributed in the visceral epithelial cells of rat glomeruli^16^, and a recent study has reported that networking and anchoring through plectin is a key to IF functionality and mechano-transduction^17^. Studies have shown several cell biological features common to podocytes and neurons: (1) long and short cell processes equipped with highly organized cytoskeletal systems; (2) cytoskeletal segregation (MTs and IFs in both cells, with an abundance of AFs); (3) process formation positively regulated by PP2A, a serine/threonine protein phosphatase; and (4) process formation accelerated by laminin^18–21^. In addition to synaptopodin, dendrin and other proteins are expressed in both neurons and podocytes. The present study also identified a novel IF protein, NEFH, in podocytes, which is commonly expressed in the nervous system.

The expression and functions of the IFs vimentin, nestin, and desmin have been extensively demonstrated in podocytes; however, NEFH has not previously been reported. Vimentin has been reported to be a mesoderm-specific marker present especially at the edge of cells with focal contacts^21^; and Tsuruta *et al*.^22^ demonstrated that vimentin regulates the structure of focal contacts and suggested that it is involved in the aggregation of “clusters” of focal contacts in cells. In the present study, the knockdown of NEFH diminished the size and amount of vinculin, an important molecule involved in focal contact with talin and tensin. Thus, NEFH might influence morphological changes in vinculin, thereby affecting focal contacts. We stained podocytes (*in vitro*) and glomeruli (*in vivo*) for vimentin and NEFH 3 days after ADR injury; this time point was chosen because we a previous study reported that podocyte injury starts on day 3 with dendrin expression and apoptotic changes^3^. In podocytes, the NEFH and vinculin staining showed similar patterns and closely colocalized; however, in the ADR-injured glomeruli, there was only partial overlap. Marvin *et al*.^23^ demonstrated that vimentin and desmin (type III IFs) can form heterodimers and heterotrimers, and it is likely that nestin, vimentin, and desmin co-assemble into mixed polymers in podocytes. It is possible that NEFH forms heterodimers with other IFs in podocytes in the same way.

Nestin was first reported to be expressed in central nervous system stem cells^24^. The stable nestin expression in cells obtained from human adults is considered a rare phenomenon and a marker of stem or progenitor cells^24,25^. Nestin is expressed in the human kidney from the first steps of glomerulogenesis within podocytes, mesangial cells, and endothelial cells^26^. This expression, restricted to podocytes in mature glomeruli, appears to be associated with cyclin-dependent kinase 5. Following differentiation, nestin is downregulated and replaced by other IFs; however, it is re-expressed following injury and in regeneration phenomena^27,28^. According to these reports, the NEFH expression appears to follow a pattern similar to that of nestin. In the present study, we double-stained for nestin and NEFH in podocytes (*in vitro*) and in glomeruli 3 days after ADR-induced injury (*in vivo*), similar to the staining for vimentin. NEFH and nestin showed a similar pattern of staining (with strong overlap) in the cultured podocytes, but only partial overlap in the ADR-injured glomeruli. As mentioned above, NEFH may co-assemble with nestin to form heterodimer filaments.

The dysfunction and loss of podocytes are hallmarks of progressive glomerular diseases. Several studies on the actin microfilament system have promised to improve the understanding of the mechanisms of podocyte injury; however, little is known about IFs. The present study provided evidence of NEFH upregulation in human FSGS and MN and showed that NEFH is a novel IF that helps protect podocytes in glomerular diseases. It is difficult to explain why the kidney samples from patients with MCD did not show a marked increase in NEFH expression; however, possible explanations may include the following: (1) the increased NEFH expression may be related to the severity of glomerular lesions, (2) podocytes may not seriously be injured in the early stage of MCD, and (3) NEFH may not be involved in MCD. Further research involving a larger number of cases with more clinical and pathological features is needed to investigate these possibilities and clarify the upstream signaling of NEFH.

We intended to use NEFH-KO mice to confirm our results about the protective role of NEFH in the ADR-treatment model. However, we were unable to obtain or generate these mice, which is a limitation of this study.

In this study, we focused on the role of NEFH in cytoskeletal arrangement in podocytes. Surprisingly, podocytes with overexpressed NEFH showed thick and strong bundles of actin, and we measured the interaction between NEFH and synaptopodin. Indeed, synaptopodin was well preserved in podocytes with overexpressed NEFH but not in NEFH-KD podocytes. These results suggest that NEFH can influence actin-associated cytoskeletal function via stabilized synaptopodin expression.

In conclusion, we identified NEFH as a novel protector against podocyte injury in an ADR-induced experimental model and in human FSGS and MN. NEFH alternation affected podocyte morphology and structure and might be involved in the regulation of synaptopodin expression.

## Methods

### Cell culture and ADR treatment

Conditionally immortalized mouse podocytes were cultured as previously described^29^. Briefly, undifferentiated podocytes were cultured in RPMI1640 medium (Sigma-Aldrich, Tokyo, Japan) with 10% fetal bovine serum (FBS), 100-units/ml penicillin and streptomycin (Pen/Strep; Life Technologies, Carlsbad, CA, USA), and 10-U/ml γ-interferon (IFN) at 33 °C. For differentiation, the podocytes were transferred to non-permissive conditions at 37 °C in the absence of γ-IFN and grown for 7–14 days. To detect the reaction of ADR (doxorubicin hydrochloride; Wako, Osaka, Japan), 0.25-μg/ml ADR was added to the differentiated podocytes in a regular medium for the indicated periods.

HEK293T cells were cultured in Dulbecco’s Modified Eagle Medium (Sigma) containing 10% FBS and 100-units/ml Pen/Strep. Transient transfection of HEK293T cells was performed using Fugene 6 reagent (Roche Diagnostics, Basel, Switzerland) at a DNA-to-Fugene ratio of 1:3, according to the manufacturer’s protocol.

### The ADR-induced nephrosis mouse model and isolation of the glomeruli

This study was approved by the Institutional Review Board of Juntendo University and was conducted in accordance with the Guide for the Care and Use of Laboratory Animals. Six-week-old female BALB/c mice (Oriental Yeast Co., Ltd. Tokyo, Japan) weighing approximately 20 g were housed under specific pathogen-free conditions and allowed free access to food and water. ADR was injected once via the tail vein at a dose of 10-mg/kg body weight^4^. Age-matched control mice were injected with an equal volume of phosphate-buffered serum (PBS; pH 7.0). On the indicated days, the mice were sacrificed, and the kidney tissues were obtained for immunohistochemistry after 4% paraformaldehyde (PFA) perfusion fixation.

To evaluate the NEFH protein expression, glomeruli were isolated using a modification of a method reported previously^30^. Briefly, mice kidneys were perfused via the heart with Hanks’ balanced salt solution containing 1-mg/ml iron powder, minced into approximately 1-mm^3^ pieces and digested with 1-mg/ml collagenase A and 100-U/ml DNase I diluted in Hanks’ balanced salt solution for 30 min at 37 °C. After digestion, the tissues were pressed through 100-µm and 70-µm cell strainers in turn. Finally, the iron powder-containing glomeruli were isolated using a magnetic particle concentrator. All procedures were performed on ice to prevent digestion.

### Microarray assay

A microarray assay was designed to examine the effects of ADR on the cultured podocytes as follows: (1) ADR groups: differentiated podocytes treated with 0.25-μg/ml ADR for 1, 2, 6, and 24 h; and (2) control group: differentiated podocytes without ADR treatment. Total RNA was extracted from both groups, and a microarray assay was performed for each time point using Bio Matrix Research microarray kit (Bio Matrix Research, Inc. Technical Development Center, Chiba, Japan). The microarray data were analyzed using Genespring GX software (Agilent, Santa Clara, CA, USA).

### RNA extraction and real-time PCR

Total RNA was extracted from the cultured podocytes with or without ADR treatment at the indicated time points using RNeasy Mini Kit (Qiagen, Hilden, Germany). cDNA was synthesized from 1 μg of total RNA using Random Decamers (Ambion, Austin, TX, USA) and reverse transcriptase (M-MLV; Invitrogen Life Technologies, Carlsbad, CA, USA). Real-time PCR was performed using TaqMan Fast Advanced Master Assay NEFH (Assay ID: Mm01191456_m1), glyceraldehyde-3-phosphate dehydrogenase (GAPDH, Assay ID: Mm99999915_g1), and a 7500 real-time PCR system (Applied Biosystems, Life Technologies), following the manufacturer’s instructions. All measured values were normalized with GAPDH and calculated using the comparative CT (∆∆CT) method.

### Western blot analysis and immunoprecipitation

To evaluate the protein expression in cultured podocytes and mice glomeruli, whole-cell extract samples were prepared using 1% Triton X-100 lysis buffer or 0.5% CHAPS buffer, with complete Mini Protease Inhibitor (Roche, Berlin, Germany) and PhosSTOP Phosphatase Inhibitor (Roche). The amounts of protein were measured with the Pierce 660-nm protein assay system (Thermo Scientific, Waltham, MA, USA) using albumin (Thermo Scientific) as a standard. Whole-cell extracts were separated using SDS-PAGE, transferred onto a PVDF membrane (Millipore, Darmstadt, Germany), and exposed to the following specific antibodies: anti-NEFH antibody (rabbit polyclonal antibody; Millipore; 1:500) and anti-synaptopodin antibody (mouse monoclonal antibody; Progen, Heiderberg, Germany; 1:100). Anti-GAPDH antibody (mouse monoclonal antibody; Sigma-Aldrich; 1:10000) or anti-beta-actin antibody (mouse monoclonal antibody; Sigma-Aldrich; 1: 5000) was used as an internal control. The Super Signal Western Dura reagent (Thermo Scientific) was used for detection, and LAS4000 was used to visualize the bands.

To investigate the interaction between NEFH and synaptopodin, HEK293T cells were co-transfected with Flag-control and GFP–NEFH, Flag-synpo-long and GFP-synpo-long, or Flag-synpo-long and GFP-NEFH separately. A co-immunoprecipitation study was performed as previously described^31^, with some modification. In brief, after 72 h of transfection, double-transfected HEK cells were harvested on ice using cold PBS with 50 mM of EDTA and then pelleted with centrifugation at 2000 rpm for 5 min at 4 °C. For the cell lysate, the pellet was resuspended in 1 ml of immunoprecipitation (IP) buffer [50 mM Tris (pH 7.5), 150 mM NaCl, 1.0% Triton X100] with protease inhibitors and phosphatase inhibitors and was incubated on ice for 30 min. The cell lysate was cleared with centrifugation at 14,000 rpm for 5 min, and 1 ml of cell extract was incubated overnight on a rotator at 4 °C with 50 μl of agarose beads coated with anti-FLAG-M2 antibody (Sigma-Aldrich). The beads were collected with centrifugation at 1,000 × *g* for 1 min and washed three times with 1 ml of IP buffer for 30 min on a rotator. The bound proteins were eluted by boiling the agarose beads in 50 μl of loading buffer at 95 °C for 5 min and analyzed by immunoblotting using an anti-GFP antibody (Abcam, Cambridge, UK).

For an additional co-immunoprecipitation assay for the GFP beads, we used GFP-Trap beads (ChromoTek, Hauppauge, NY, USA). As described earlier, we used HEK293T cells and co-transfected Flag-Synpo-long and GFP-vector (negative control), GFP-synpo-long (positive control), or GFP-NEFH (sample). IP was performed with GFP beads, and western blotting with was performed with Flag and GFP antibodies.

### Immunofluorescence

Mice kidneys were perfusion-fixed with 4% PFA and 20% sucrose in PBS, placed in OCT Compound, frozen in liquid nitrogen, and stored at −80 °C. Subsequently, 3-μm-thick sections were incubated overnight at 4 °C with rabbit anti-NEFH antibody (1:500), and double stained for synaptopodin (1:10) and post-stained for DAPI for the nuclei. NEFH was also double stained with α-tubulin (1:100; Oncogene Research Products, San Diego, CA, USA) and with vimentin (1:100; Abcam, Cambridge, UK), nestin (1:200; Abcam), or desmin (1:40; Sigma-Aldrich).

Human biopsy specimens were fixed with −20 °C acetone and were immunostained for NEFH, synaptopodin, and DAPI, as described earlier.

To investigate the subcellular distribution of proteins, the differentiated podocytes were cultured on collagen type I-coated cover glass, treated for 24 h with 0.25-μg/ml ADR, and then fixed with 2% PFA and 4% sucrose in PBS. The fixed cells were permeabilized with 0.3% Triton in PBS. After treating with a blocking solution (2% fetal calf serum, 2% bovine serum albumin, and 0.2% fish gelatin in PBS), the cells were incubated with the following specific antibodies: rabbit anti-NEFH antibody (1:500, at 4 °C overnight), mouse anti-synaptopodin antibody (1:10, 1 h at room temperature), and mouse anti-vinculin antibody (monoclonal antibody; Abcam; 1:200, 1 h at room temperature). Finally, the cells were incubated with donkey anti-rabbit IgG conjugated with Alexa 488 or goat anti-mouse IgG conjugated with Alexa 555 as a secondary antibody. DAPI staining for nuclei was performed. For the AF staining, Alexa Fluo555-phalloidin (Invitrogen; 1:250) was used. All images were captured using a confocal laser microscope (Olympus FV1000; Olympus Corp., Tokyo, Japan). For the quantification study, Image-pro Plus software (Media Cybernetics, Rockville, MD, USA) was used to analyze the fluorescence intensity and count the number of positive staining results.

Because of the weak immunofluorescence intensity of GFP itself, anti-GFP antibody (Clontech, Japan; 1:100) was used together with anti-NEFH antibody for the GFP–NEFH-transfected podocytes to confirm the generation of NEFH-OV podocytes.

### Plasmid constructs and transfection

The EGFP–NEFH construct was kindly provided by Dr. Aidong Yuan (Center for Dementia Research, Nathan Kline Institute, New York University School of Medicine, NY, USA). The pSuper RNAi system was used to construct NEFH siRNA, according to the manufacturer’s protocol. Three different 19-nucleotide (nt) sequences were separately inserted into the pSuper.Zeocin vector after digestion with BglII and HindIII (Promega Corp., Madison, WI, USA). These corresponded to the nucleotides GGCCAAATCTCCAGCTGAA, CCTCTAGATGTGAAGTCT, and GGAGAGTAAGAAAGACGAA of mouse NEFH mRNA; they were separated from the reverse complement of the same 19-nt sequence by a 9-nt non-complementary spacer (TTCAAGAGA). A control vector with a scramble sequence, pSup.cont., was produced as described previously^31^. All plasmids were confirmed using DNA sequencing.

To establish a stable NEFH-KD podocyte cell line, confirmed NEFH siRNA plasmids were transfected to undifferentiated podocytes. Stable transfecting selection was performed with 500-μg/ml zeocin (Invitrogen), starting 48 h after transfection. Stable transfected clonal cell lines were produced and cultured with a maintenance concentration of 200-μg/ml zeocin.

To establish a NEFH-OV podocyte cell line, undifferentiated podocytes were transfected with 1 μg of the EGFP–NEFH construct and selected with 1000-μg/ml G418 (Roche). NEFH expression in the NEFH-KD and NEFH-OV podocytes was tested using western blotting, as described earlier.

### Adhesion assay

To examine the effect of gene interference of NEFH on the cellular adhesive property of podocytes, differentiated podocytes were trypsinized, seeded at 10^4^ cells per well in a collagen type I-coated 96-well plate, and continuously incubated for 2 h at 37 °C. Cells that did not adhere were removed carefully with PBS. Adhesive cells were fixed with cold methanol for 10 min and stained with 0.1% crystal violet for 15 min at room temperature. After washing out the staining dye, the cells were lysed with 1% SDS and the optical density (OD) value at 570 nm was examined. Cells in nine culture wells were analyzed for each condition.

### Detachment assay

To investigate ADR-induced podocyte detachment, the same number of differentiated cells cultured in the 96-well plate were stimulated with 1-μg/ml ADR for 20 h. After removing the detached cells using PBS, the cells were fixed and stained with 0.1% crystal violet, and the OD value at 570 nm was examined, as described above. The percentage of detached cells was calculated using the following formula: (OD value of untreated cells − OD value of undetached cells)/OD value of untreated cells × 100. Cells in nine culture wells for each condition were analyzed.

### Human biopsy samples

Human kidney tissue samples were obtained from diagnostic renal biopsies performed at Juntendo University Hospital. This experiment was conducted according to the principles of the Declaration of Helsinki and was approved by the Institutional Review Board of Juntendo University Hospital. Written informed consent was obtained from all patients. To assess NEFH expression in human podocytes, triple staining for NEFH, synaptopodin, and DAPI was performed on kidney biopsy samples from patients diagnosed with MCD (n = 5), FSGS (n = 5), and MN (n = 7). Biopsy samples from patients with minor glomerular abnormalities (n = 7) were used as controls. The patients’ clinical data are presented in Supplementary Table 1. The immunofluorescence intensity was analyzed using Image-pro Plus software (Media Cybernetics).

### Statistics

Data are presented as mean ± SE. All experiments were repeated at least three times, and representative experiments are presented. To evaluate differences between groups, Student’s *t*-test was used for comparisons of two groups and ANOVA, followed by the LSD test, was used for comparisons of more than two groups. A P-value of <0.05 was regarded as statistically significant. All analyses were performed using SPSS Statistics 17.0 (IBM Corp., Armonk, NY, USA).

## Electronic supplementary material

Supplementary Figure 1 and Supplementary Table 1
