# Supplementary material for: Neurofilament heavy polypeptide protects against reduction in synaptopodin expression and prevents podocyte detachment
Source: Sci Rep. 2018 Nov 21;8:17157. doi: 10.1038/s41598-018-35465-6 (PMC6249220; doi:10.1038/s41598-018-35465-6)
Supplement: Supplementary file 1 — Supplementary Figure 1 and Supplementary Table 1 [file 41598_2018_35465_MOESM1_ESM.pdf]

Neurofilament heavy polypeptide protects against reduction in synaptopodin expression and prevents podocyte detachment

Juan Wang, Teruo Hidaka, Yu Sasaki, Eriko Tanaka, Miyuki Takagi, Terumi Shibata, Ayano Kubo, Juan Alejandro Oliva Trejo, Lining Wang, Katsuhiko Asanuma, and Yasuhiko Tomino

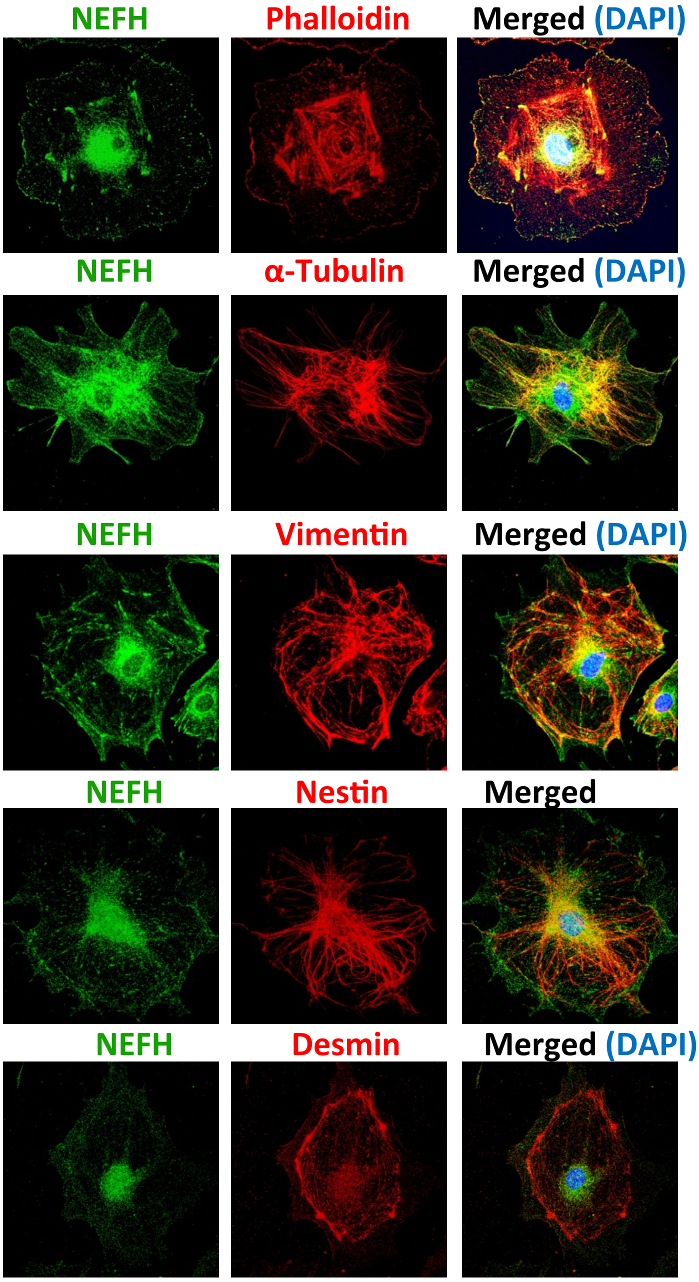

Supplementary Figure 1. Neurofilament heavy polypeptide (NEFH) was not colocalized with phalloidin, tubulin, vimentin, nestin, or desmin

As controls, wild-type podocytes were stained for NEFH and phalloidin, tubulin, vimentin, nestin, or desmin. Under normal conditions, the NEFH expression was weak and localized mainly around the nuclei.

Clinical data associated with the human kidney samples

Diagnosis	Symptom	Age (years)	u-pro	u-bld	sCre (mg/dl)	eGFR
MCD	NS	38	26 g/day	1-4/HPF	1	65
MCD	NS	75	7.83 g/day	1-4/HPF	1.1	50
MCD	NS	17	6 g/g.Cr	1-4/HPF	0.6	110
MCD	NS	29	22 g/day	1-4/HPF	1.1	70
MCD	NS	20	9 g/day	1-4/HPF	0.6	130
MN	NS	46	11 g/g.Cr	1-4/HPF	0.9	72
MN	NS	43	3.7 g/g.Cr	1-4/HPF	0.7	97
MN	NS	60	9.8 g/g.Cr	5-9/HPF	0.7	65
MN	NS	68	17 g/g.Cr	11-15/HPF	1	60
MN	NS	74	12 g/g.Cr	6-10/HPF	0.65	65
MN	NS	54	7.9 g/day	1-5/HPF	0.85	75
MN	NS	67	8.6 g/g.Cr	1-5/HPF	0.7	60
FSGS	NS	57	9.6 g/day	6-10/HPF	1	60
FSGS	NS	26	7.1 g/g.Cr	6-10/HPF	0.7	80
FSGS	NS	47	6.5 g/g.Cr	6-10/HPF	0.6	100
FSGS	CGN	16	1.1 g/g.Cr	30-49/HPF	0.6	140
FSGS	CGN	46	3.6 g/g.Cr	11-15/HPF	0.8	80

MCD: minimal change disease; MN: membranous nephropathy; FSGS: focal segmental glomerulosclerosis; NS: nephrotic syndrome; CGN: chronic glomerulonephritis; u-pro: urine protein; u-bld: urine blood; sCre: serum creatinine; eGFR: estimated glomerular filtration rate

Supplementary Table 1. Clinical data associated with the human kidney biopsy samples

The kidney biopsy samples were diagnosed by pathological specialists.
